# Characterization of Pb51 in *Plasmodium berghei* as a malaria vaccine candidate targeting both asexual erythrocytic proliferation and transmission

**DOI:** 10.1186/s12936-017-2107-2

**Published:** 2017-11-13

**Authors:** Jian Wang, Wenqi Zheng, Fei Liu, Yaru Wang, Yiwen He, Li Zheng, Qi Fan, Enjie Luo, Yaming Cao, Liwang Cui

**Affiliations:** 1 Department of Immunology, College of Basic Medical Sciences, China Medical University, Shenyang, 110001 Liaoning China; 2 Laboratory of Surgery, The Affiliated Hospital, Inner Mongolia Medical University, Hohhot, 010050 China; 3 Dalian Institute of Biotechnology, Dalian, Liaoning China; 4 Department of Pathogen Biology, College of Basic Medical Sciences, China Medical University, Shenyang, 110001 Liaoning China; 5 Department of Entomology, Pennsylvania State University, University Park, PA 16802 USA

**Keywords:** *Plasmodium berghei*, Pb51, Vaccine candidate, Asexual blood stage, Sexual stage, Transmission-blocking

## Abstract

**Background:**

A vaccine that targets multiple developmental stages of malaria parasites would be an effective tool for malaria control and elimination.

**Methods:**

A conserved gene in *Plasmodium,* the *Plasmodium berghei* gene (*PBANKA_020570*) encoding a 51 kDa protein (*pb51* gene), was identified through search of the PlasmoDB database using a combination of expression and protein localization criteria. A partial domain of the Pb51 protein was expressed in a prokaryotic expression system (rPb51) and used for immunization in mice. The protein expression profile and localization were studied by Western blot and indirect immunofluorescence assay (IFA), respectively. The inhibitory effect of the anti-rPb51 antibodies on parasite proliferation was evaluated in erythrocytes in vivo. The transmission-blocking activity of the immune sera was determined by in vitro ookinete conversion assay and by direct mosquito feeding assay (DFA).

**Results:**

The rPb51 elicited specific antibodies in mice. Western blot confirmed Pb51 expression in schizonts, gametocytes and ookinetes. IFA showed localization of Pb51 on the outer membranes of schizonts, gametocytes, zygotes, retorts, ookinetes and sporozoites of *P. berghei.* Mice immunized with the rPb51 protein significantly reduced parasite proliferation and gametocyte conversion in vivo. Moreover, the rPb51 antisera also significantly reduced the in vitro ookinete conversion when added into the ookinete culture medium. In DFA, mice immunized with the rPb51 reduced the prevalence of mosquito infection by 21.3% and oocyst density by 54.8%.

**Conclusions:**

In *P. berghei*, P51 was expressed in both asexual erythrocytic and sexual stages and localized on the surface of these stages with the exception of the ring stage. The anti-rPb51 antibodies inhibited both *P. berghei* proliferation in mice and transmission of the parasite to mosquitoes.

**Electronic supplementary material:**

The online version of this article (10.1186/s12936-017-2107-2) contains supplementary material, which is available to authorized users.

## Background

Malaria remains a serious global health burden with 95 countries and territories with ongoing malaria transmission. Approximately 214 million new clinical cases and 438,000 deaths were recorded in 2015 [1]. Malaria control efforts rely heavily on treatment with artemisinin-based combination therapy (ACT), indoor residual spraying of insecticides, and insecticide-treated mosquito nets, but these measures have become less effective due to the emergence of multidrug-resistant parasites and insecticide-resistant mosquitoes [2, 3]. As many malaria-endemic nations are pursuing malaria elimination [4], these technical challenges require the development of integrated approaches, among which safe and effective malaria vaccines could be a crucial tool [5].

Three strategic approaches for malaria vaccine development target different stages of the malaria parasite life cycle [6–8]. Pre-erythrocytic vaccines targeting the sporozoites and liver stages are designed to protect residents in low-endemic areas from becoming infected. Blood-stage malaria vaccines targeting the asexual blood stages aim to induce immunity to reduce the severity of the clinical disease. Transmission-blocking vaccines (TBVs) targeting the sexual stages and mosquito midgut antigens aim at inducing immunity to interrupt malaria transmission. Currently, the pre-erythrocytic sub-unit vaccines have concentrated on the circumsporozoite protein (CSP). The leading liver-stage vaccine RTS,S, which can induce CD4^+^ T cell and antibody responses against CSP [9], has only shown partial protection against clinical malaria [10, 11]. Blood-stage malaria vaccines have focused on merozoite antigens, such as the apical membrane antigen 1 and merozoite surface proteins, that are involved in the invasion of erythrocytes [12, 13]. TBVs have primarily targeted a few candidates expressed on gametocytes and gametes such as P48/45 [14, 15] and P230 [16, 17], as well as those on zygotes and ookinetes such as P25 and P28 [18–20]. Given the complex life cycle of malaria parasites, malaria vaccines should ideally target multiple developmental stages and multiple malaria parasite species.

A vaccine that targets both asexual blood stages and sexual stages would not only offer direct protection against clinical disease, but also have the benefit of reducing transmission [21]. However, the majority of the vaccine candidates in the development pipeline are stage-specific; single vaccines providing broad and sustained protection against different stages are notably deficient. The extensive ‘omics’ data on all stages of the entire malaria parasite life cycle offer an unprecedented opportunity for a reverse vaccinology approach to systematically in silico search novel vaccine candidates with desired expression properties [22].

By searching the omics data in PlasmoDB database [23] using defined criteria, a conserved *Plasmodium* protein that contains a signal peptide and an OST3_OST6-like domain was identified. This domain is described in the PFAM database as a domain present in the transporter protein family necessary for N-glycosylation. This protein is presumably expressed in both asexual blood stages and sexual stages based on available transcriptomic data. This gene in the rodent malaria parasite *Plasmodium berghei* encodes a hypothetical 51-kDa protein, and is thus referred to as Pb51. In this study, the protein expression profile of Pb51 in *P. berghei* was examined, and its potential as a vaccine targeting both blood stage and parasite transmission was evaluated.

## Methods

### Sequence analysis

The PlasmoDB database was searched using a combination of criteria including the presence of a signal peptide, two or more transmembrane domains, expression in both blood stages and gametocytes in *P. falciparum*, red blood cell (RBC) targeting with the presence of a PEXEL motif, and conservation among *Plasmodium* species. The genomic sequences of *p51,* a gene identified from this search, were retrieved from multiple *Plasmodium* species in PlasmoDB. Multiple sequence alignment was performed using ClustalW. Domain organization of the encoded proteins was predicated using the simple modular architecture research tool as described previously [23].

### Animals and parasite

Six- to 8-weeks-old female BALB/c mice and New Zealand White (NZW) female rabbits (Beijing Animal Institute, China) were used following the guidelines approved by the animal ethics committee of China Medical University. The *P. berghei* ANKA strain 2.34 was maintained in BALB/c mice with passages through adult *Anopheles stephensi* as described previously [23]. For initiating a blood-stage infection, 1 × 10^6^ *P. berghei*-infected RBCs (iRBCs) were injected intraperitoneally (ip) into each mouse. Parasitaemia was measured daily by microscopy of Giemsa-stained blood smears.

### Production of recombinant protein

**Fig. 1:**
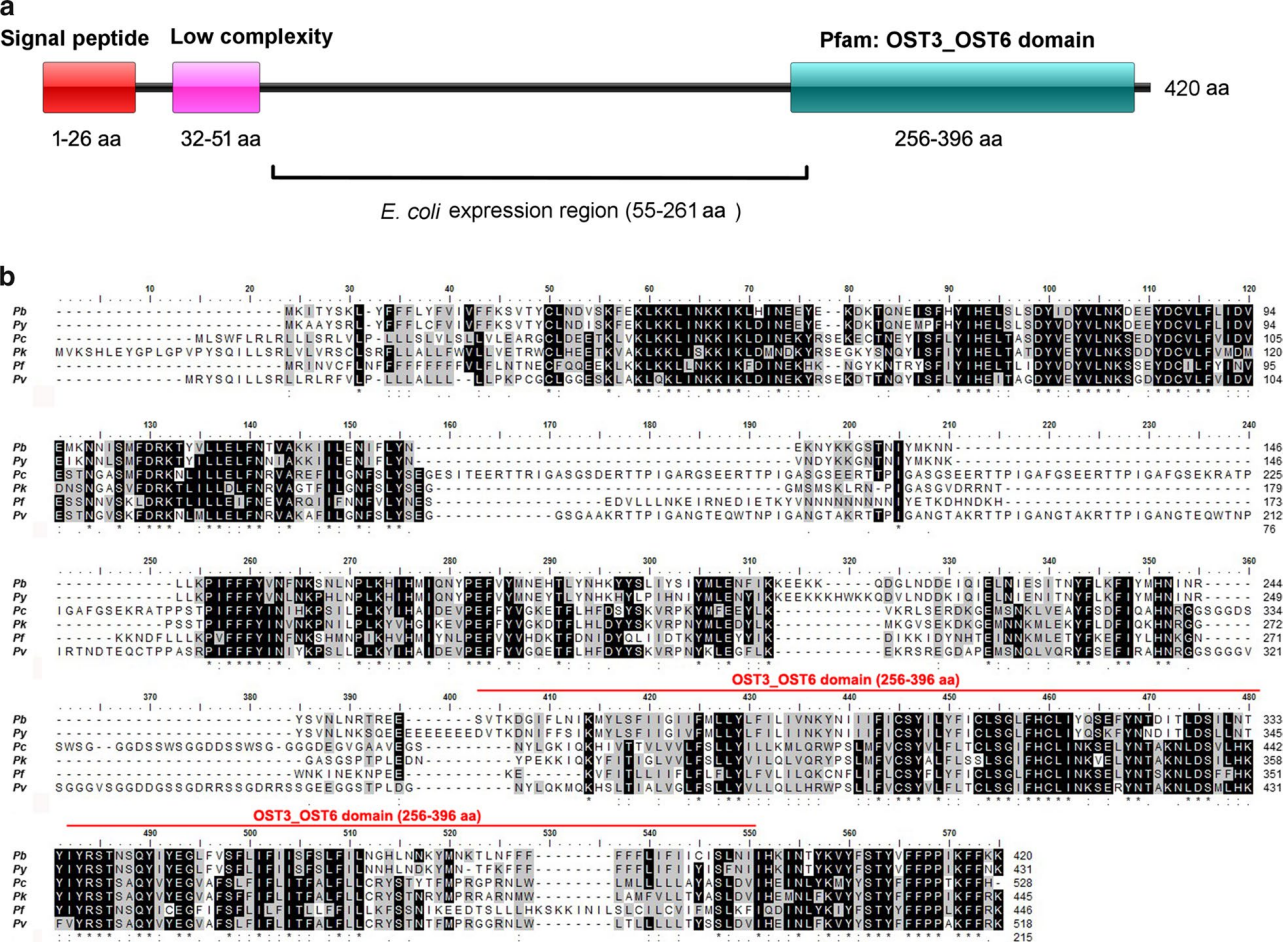
Sequence analysis and schematic domain composition of Pb51. **a** Schematic domain organization of Pb51. The signal peptide, low complexity region and the OST3_OST6-like domain are shown as coloured boxes. The fragment used for *E. coli* expression is also marked. **b** Alignment of P51 orthologs in *Plasmodium* species. Pb (*P. berghei*), Py (*P. yoelii*), Pc (*P. chabaudi*), Pk (*P. knowlesi*), Pf (*P. falciparum*), Pv (*P. vivax*). Conserved amino acids are shadowed in black (for identical residues) and grey (for similar residues). The OST3_OST6-like domain is highlighted. The predicted PEXEL motif is located at aa 38–44 of the Pf51 sequence

A 207-amino acid (aa) fragment (aa 55–261) of *pb51*, excluding the putative signal peptide, low-complexity region and transmembrane domains, was used for recombinant Pb51 protein (rPb51) expression (Fig. 1a). The *pb51* fragment was PCR amplified using *P. berghei* genomic DNA as the template with a sense primer *pb51*F (5′-CTGGATCCGATAAAACACAAAATGAAATATCATT-3′, *Bam*HI site underlined) and a reverse primer *pb51*R (5′-CAGCGGCCGCACCATCTTTAGTTACAGATTCTTC-3′, *Not*I site underlined) and cloned into the prokaryotic expression vector pET32a (+). The recombinant Pb51 protein (rPb51) protein was expressed in *Escherichia coli* Rosetta-gami B (DE3) (Novagen) as a fusion protein with a Trx/His/S-tag and purified using Ni-NTA His-Bind Superflow resin as described previously [24]. The Trx/His/S-tag without Pb51 protein in the expression vector pET32a (+) was also purified as a control for immunization. The purified protein was analysed by SDS-PAGE on 10% gels under reducing conditions. Protein concentration was determined by using a BCA Protein Assay Kit (Thermo Scientific).

### Immunization scheme and antibody quantification

Two groups of 6–8 weeks-old, female BALB/c mice (six per group) were used for immunization with the rPb51 or Trx/His/S-tag protein. Each mouse was immunized subcutaneously with 50 μg of protein emulsified in 100 μl of complete Freund’s adjuvant (Sigma) at primary immunization. At 15 and 30 days after the first immunization, mice were boosted with 25 μg recombinant protein emulsified in incomplete Freund’s adjuvant (Sigma). Serum was collected from the tail vein of mice before each immunization and at 10 days after the last immunization to assess the antibody response. The antisera from the final collection were pooled by immunization groups.

To produce antibodies against Pb51 in rabbits, two groups of 3–4 months old New Zealand White rabbits (two per group) were immunized subcutaneously with 100 μg of rPb51 formulated with 100 μl of complete Freund’s adjuvant followed by two booster immunizations with 50 μg recombinant protein emulsified in incomplete Freund’s adjuvant at 15 and 30 days after the first immunization. Antisera were collected 10 days after the last immunization. Anti-rPb51 antibodies in immunized mice and rabbits were quantified by enzyme-linked immune sorbent assay (ELISA) essentially as described earlier [24].

### Western blot

The purification or enrichment of schizonts, gametocytes and ookinetes was performed as previously described [24]. After purification, they were washed twice with phosphate-buffered saline (PBS, pH 7.0) and treated with 0.15% saponin to lyse the erythrocytes. Parasite proteins were extracted using 1% Triton X-100 and 2% SDS in PBS with 1× protease inhibitors for 30 min at room temperature. For Western blot, 10 μg of parasite proteins from each stage (schizonts, gametocytes and ookinetes) were electrophoresed on a 10% SDS-PAGE gel under reducing conditions and transferred to a 0.22-μm PVDF membrane (Bio-Rad). The membrane was blocked with 5% (w/v) skimmed milk dissolved in PBS-T (PBS with 0.05% Tween 20) for 2 h at 37 °C, and probed with the mouse anti-rPb51 antisera (1:500) for 2 h at room temperature. Following washes with PBS-T, the membrane was incubated with HRP-conjugated goat anti-mouse IgG antibodies (1:5000, Invitrogen) in blocking solution. The blot was developed and visualized using a Pierce ECL Western Blotting Kit (Thermo Scientific).

### Indirect immunofluorescence assay (IFA)

Indirect immunofluorescence assay was performed to detect Pb51 expression in asexual blood stages, gametocytes, zygotes, retorts, ookinetes and sporozoites of *P. berghei.* Parasites were air-dried on slides and then fixed with paraformaldehyde (Sigma) in PBS for 20 min. After permeabilization with 0.1% Triton X-100 (Sigma), the slide was blocked in 5% skim milk in PBS for 1 h at 37 °C. Pooled antisera against rPb51 were diluted (1:500) with 5% skim milk in PBS-T for 1 h at 37 °C. After washing with 0.1M PBS, FITC-labelled goat anti-mouse IgG (1:500, Invitrogen) was used as the secondary antibody. To co-localize Pb51 with major ookinete surface protein Pbs21, parasites were air-dried on slides and then fixed with paraformaldehyde in PBS for 20 min. Anti-rPb51 rabbit sera was used as first antibodies and detected by Alexa Fluor 555-conjugated secondary antibodies (red). Anti-Pbs21 mouse mAb was used for surface staining detected by FITC-labeled secondary antibodies (green). Parasite nuclei were stained with 4′,6-diamidino-2-phenylindole (DAPI; Invitrogen). The specimen was observed under an Olympus BX53 (Olympus Corporation) microscope.

### Active immunization, passive transfer of antisera and challenge experiments

Immunization of mice with rPb51 or the control protein was performed as described above. For passive antibody transfer, each mouse (six per group) received three daily ip injections of 125 µl anti-rPb51mouse sera, control sera, or PBS beginning on day 0 of *P. berghei* infection. Mice were infected ip with 1 × 10^6^ iRBCs as described above. The parasitaemia and the survivorship of mice were monitored daily as described above.

### Exflagellation of male gametocytes and ookinete formation inhibition assay

To examine the transmission-blocking (TB) activity of the anti-rPb51 sera, 10 μl of infected mouse blood was mixed with the 90 μl ookinete culture medium (100 mg/l neomycin, 50 mg/l streptomycin, 50 mg/l penicillin, 20% (v/v) FBS, and 1 mg/l heparin in RPMI 1640, pH 8.3) containing anti-rPb51 sera or control sera at final dilutions of 1:5, 1:10 and 1:50 and used in the male gametocyte exflagellation and ookinete formation inhibition assay as previously described [23]. Male gamete exflagellation centers were counted after incubation at 25 °C for 15 min [23], while ookinete development was enumerated after incubation at 19 °C for 24 h by fluorescence microscopy with anti-Pbs21 monoclonal antibody [25, 26]. In another experiment, rPb51-immunized or control mice were inoculated ip with 5 × 10^6^ iRBCs. On day 3 post-infection, 10 µl of parasite-infected blood from the mouse tail vein were directly added to 90 µl ookinete culture medium. At 24 h, different parasite stages during ookinete conversion were enumerated as described above.

### Direct mosquito feeding assays (DFA)

Mice (three per group) were immunized with the rPb51 or the control protein as described above, and infected ip with 5 × 10^6^ *P. berghei*-iRBCs at 10 days after the second boost. Three days after infection, they were fed with starved, 4-days-old female *An. stephensi* mosquitoes for 30 min. After removal of the unfed mosquitoes, engorged mosquitoes were maintained in an insectary at 19–21 °C and 70% relative humidity. Ten days after feeding, at least 50 mosquitoes were dissected from each group to determine the prevalence (proportion of infected mosquitoes) and intensity (number of oocysts per midgut) of infection [24].

### Statistical analysis

Statistical analysis was performed using GraphPad Prism software (version 6.01) and SPSS version 17.0. The optical density value, parasitaemia, exflagellation, and ookinete numbers were compared using the Student’s *t* test. The numbers of surviving mice between the two immunization groups were compared using the Kaplan–Meier test. The prevalence of infection was analysed by Fisher’s exact test and the intensity of infection was analysed by the Mann–Whitney *U* test. Significance was set at *P* < 0.05.

## Results

### Pb51 is a conserved *Plasmodium* protein

In order to identify potential vaccine candidates that could target both asexual erythrocytic and sexual stages, the PlasmoDB was searched using a number of criteria for expression and subcellular localization. Seven genes satisfied all criteria including three conserved hypothetical proteins, two exported proteins of unknown functions, a rifin, and CX3CL1-binding protein. A gene (PBANKA_020570) annotated as “conserved *Plasmodium* membrane protein of unknown function” for further analysis in the rodent parasite *P. berghei* was selected. This gene, designated *pb51*, encodes a protein of 420 aa with a calculated molecular weight of 51 kDa. In addition to the putative signal peptide, the ~ 140-aa C-terminal region has four predicted transmembrane helices that resemble the OST3_OST6 domain in the PFAM database (Fig. 1a). OST3 and OST6 are homologous proteins present in the oligosaccharyl transferase complex within the lumen of the rough endoplasmic reticulum, which mediates *en bloc* transfer of a high-mannose oligosaccharide moiety to asparagine acceptor sites in nascent polypeptides [27, 28]. Yet, the presence of a PEXEL motif in Pf51, which would target the protein to the RBC membrane, suggests that the OST3_OST6-like domain may have different functions in *Plasmodium* other than glycosylation. This gene is highly conserved among all *Plasmodium* species, as evidenced from the alignment of the predicted amino acid sequences (Fig. 1b).

### The rPb51 protein is immunogenic

**Fig. 2:**
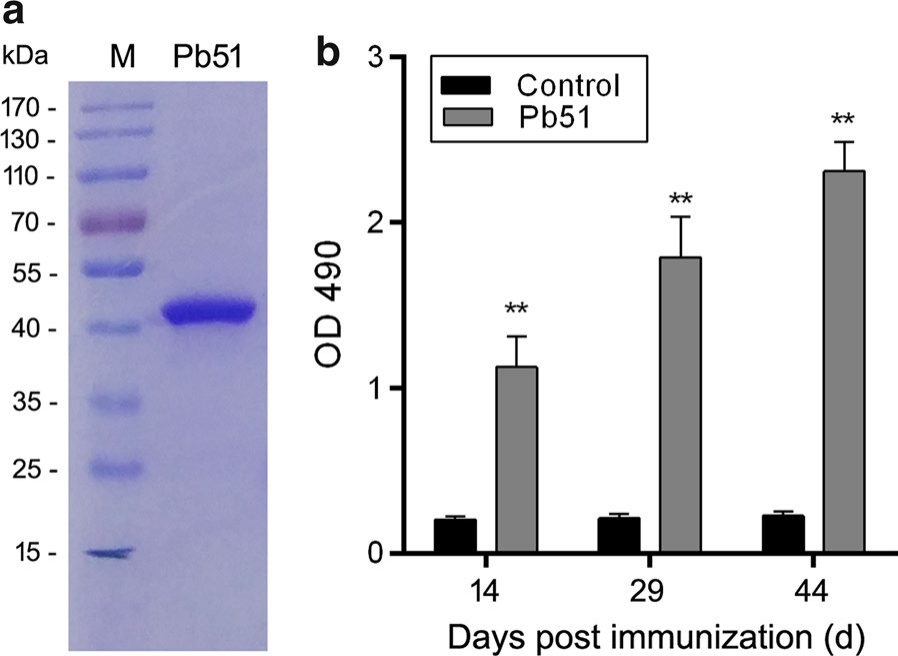
Production of rPb51 and immunization. **a** rPb51 was purified from *E. coli* and analysed by SDS-PAGE under reducing conditions. Molecular weight markers are shown on the left. **b** Anti-rPb51 antibody titres after immunizations. Serum samples were collected on days 14, 29 and 44 post-immunization. Antibody titres correspond to the last dilution of the anti-rPb51 serum, wherein OD490 values were above the cut-off values in ELISA. Cut-off value was defined as that of the pooled sera from control mice. Serum samples were tested at 1: 200–1:102,400 serial dilutions, 1:200 dilutions were used and the data represent three separate experiments. Error bars indicate mean ± SD. *P < 0.05, **P < 0.01 (Student’s *t* test)

In order to produce soluble recombinant protein in bacteria, the aa 55–261 region between the signal peptide and the first transmembrane domain of Pb51 was cloned into the expression vector (Fig. 1a). This region includes eight predicted antibody epitopes (Additional file 1: Figure S1). The recombinant protein was expressed in *E. coli* as a Trx/His/S-tag fusion protein. Protein expression was induced at low temperature (20 °C) for 12 h to enhance protein solubility. The rPb51 was present in the soluble fraction of the lysate and thus performed purification under native conditions. SDS-PAGE analysis showed that rPb51 migrated as a single band at approximately 44 kDa, consistent with the predicted molecular size of the rPb51 fusion protein (Fig. 2a).

To determine the immunogenicity of rPb51, BALB/c mice were immunized with purified rPb51 emulsified in Freund’s adjuvants to produce polyclonal antisera. IgG levels against rPb51 in mouse antisera during the course of immunization were followed using ELISA. IgG titres in the rPb51 immunization group showed statistically meaningful increases at all sampling time points (Fig. 2b, *P* < 0.01, Student *t* test), which indicates the rPb51 successfully induced the production of antibodies in mice.

### Pb51 is expressed both on asexual stages and sexual stages

The orthologue of Pb51 in *P. falciparum* (PF3D7_0107700) is expressed in both asexual erythrocytic stages [29] and mature male/female gametocytes [30, 31]. To determine the expression of Pb51 during development, Western blot analysis was performed using protein extracts obtained from purified schizonts, gametocytes and ookinetes.

**Fig. 3:**
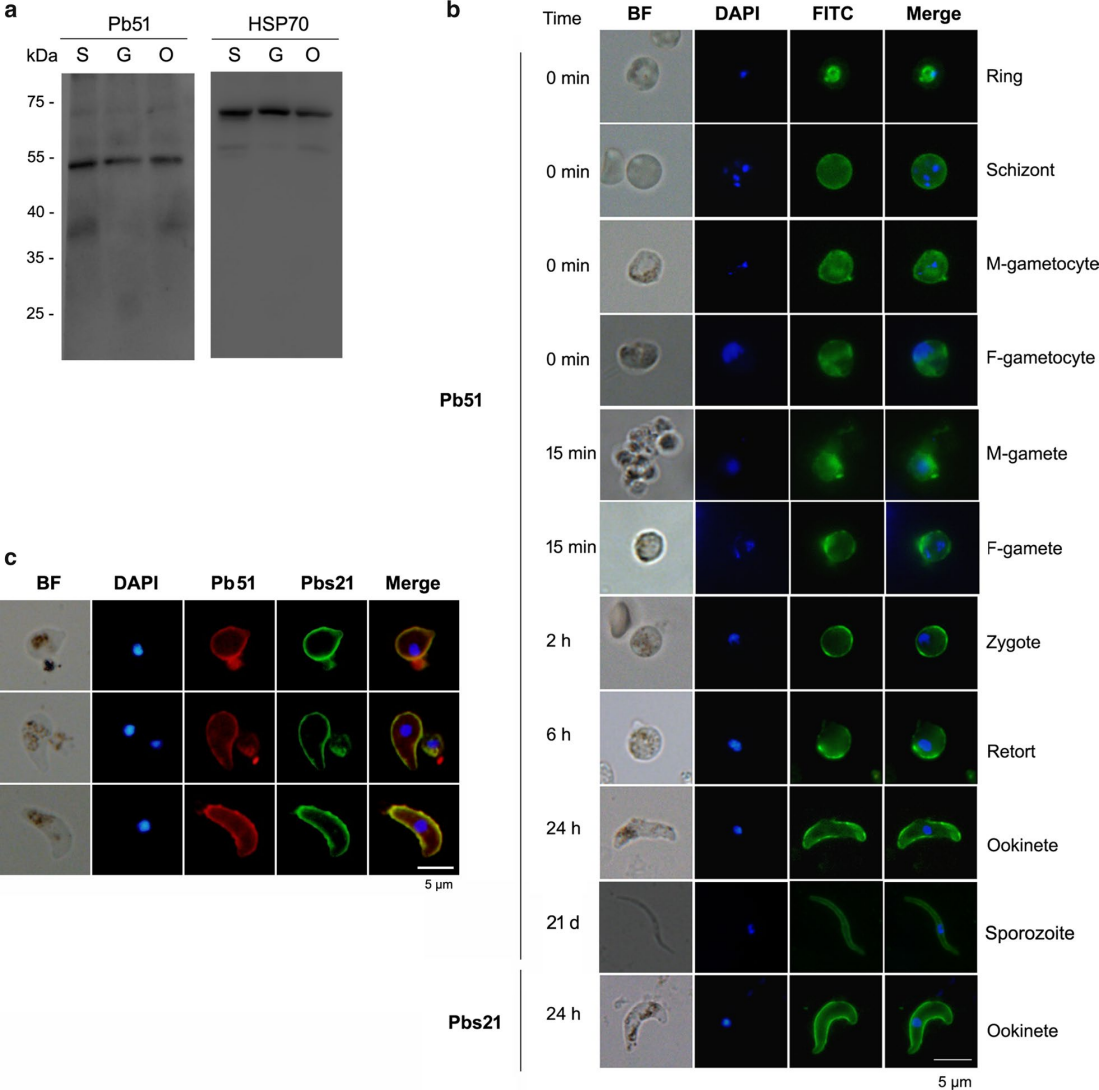
The expression profile and localization of Pb51. **a** Western blot analysis of Pb51 expression in lysates from *P. berghei* schizont (S), gametocytes (G), and ookinetes (O). Anti-HSP70 serum was used for protein loading control. **b** IFA analysis of Pb51 localization. Different stages (rings, schizonts, gametocytes, zygotes, retorts, ookinetes and sporozoites) at different time points of *P. berghei* development were used. Pb51 was detected by FITC-conjugated secondary antibodies (green). Cells were permeabilized with 0.1% Triton X-100. Pbs21 mAb was used for surface staining of ookinetes. *BF* bright field. **c** Co-localization IFA analysis of Pb51 expression. Retorts and ookinetes were proceeded directly for antibody binding. Anti-rPb51 rabbit sera was used as first antibodies and detected by Alexa Fluor 555-conjugated secondary antibodies (red). Anti-Pbs21 mouse mAb was used for surface staining detected by FITC-labeled secondary antibodies (green). For Fig. 3b, c, parasite nuclei were stained with DAPI (blue). The scale bar indicates 5 µm

The anti-rPb51 sera recognized a band of approximately 51 kDa in the lysates of all parasite stages tested, which is close to the predicted size of Pb51 (Fig. 3a). Then, the cellular locations of Pb51 using IFA was examined. Consistent with the results from the Western blot, the pooled antisera against rPb51 at 500-fold dilution successfully stained all *P. berghei* stages examined (rings, schizonts, gametocytes, gametes, zygotes, retorts, ookinetes and sporozoites) (Fig. 3b). The results indicated the anti-rPb51 could recognize the native parasite antigens. Except for the ring stage where fluorescence was restricted to the parasite inside the iRBC, IFA with anti-rPb51 antisera all showed fluorescent patterns that are consistent with surface staining (Fig. 3b). In retorts and ookinetes, the staining with anti-rPb51 well overlapped with that of Pbs21 (Fig. 3c). Moreover, IFA with or without membrane permeabilization showed similar fluorescence patterns (Additional file 2: Figure S2), indicating that the Pb51 protein is localized on the outer surfaces of both asexual and sexual stages as well as sporozoites of *P. berghei.*

### Immunization with rPb51 protects against infection

**Fig. 4:**
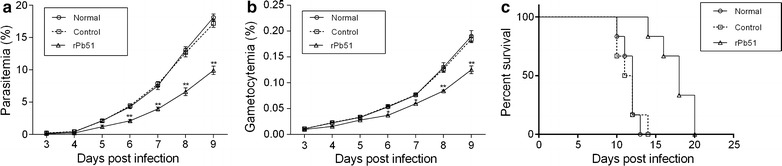
Effects of immunization against rPb51 on asexual proliferation, gametocytogenesis and host survival. **a** Growth curves of *P. berghei* in normal BALB/c mice (no immunization) and mice immunized with the control Trx/His/S-tag protein (control) or rPb51. Normal group exhibited 1.82-fold higher parasitaemia than the rPb51-immunized group on day 9 post-infection. **b** Gametocytaemia in mice without immunization (normal), immunized with Trx/His/S-tag protein (control) or rPb51. Note that gametocytaemia on day 3 was not statistically different among the immunization groups. **c** Survival of mice in different treatment groups. Mice in the rPb51-immunized group survived 6 days longer than the normal group and 7 days longer than control group. The data represent three separate experiments (six mice/group). Error bars indicate mean ± SD. *P < 0.05, **P < 0.01 (Student’s *t* test)

Given the localization of Pb51 on the surface of later stages of asexual erythrocytic cycle, whether the antibodies against this protein affect asexual erythrocytic development was determined. To do this, mice were immunized with the rPb51 protein emulsified in complete Freund’s adjuvant, and after two boosts with rPb51 protein emulsified in incomplete Freund’s adjuvant, they were challenged by ip injection with 1 × 10^6^ *P. berghei*-iRBCs. It was evident that immunization with rPb51 greatly slowed down the rise of both asexual parasitaemia (Fig. 4a, *P* < 0.01, Student *t* test) at 6–9 days post infection, and gametocytaemia (Fig. 4b, *P* < 0.01, Student *t* test) at 8 and 9 days post-infection, and delayed the death of the infected mice (Fig. 4c, *P* < 0.01, Kaplan–Meier test). In addition, *P. berghei* infected mice passively treated with three daily transfers of the anti-rPb51 sera also showed significant inhibition of parasite development and better survivorship of the infected mice (Additional file 3: Figure S3). These data collectively indicate that anti-rPb51 antibodies provide some degree of protection against *P. berghei* infection in mice.

### Antibodies against Pb51 show obvious TB activities

**Fig. 5:**
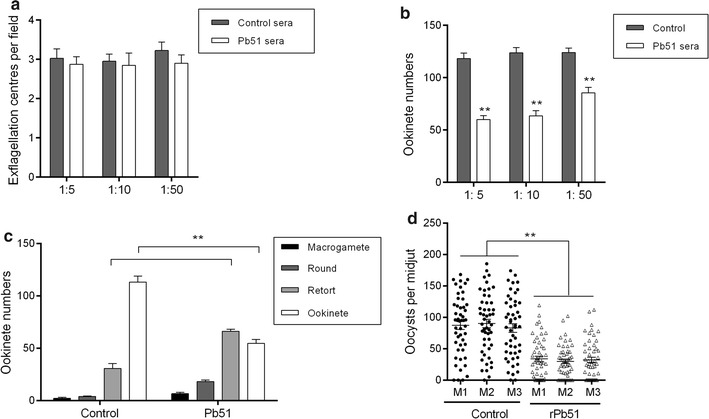
TB activities of the anti-rPb51 sera. **a** Effect of the antiserum on exflagellation of male gametocytes. Anti-rPb51 sera, or control mouse sera were diluted at 1:5, 1:10 and 1:50 and incubated with gametocytes to quantify exflagellation centres. **b** Effect of anti-rPb51 sera at 1:5, 1:10 and 1:50 dilutions on *P. berghei* ookinete formation in vitro. **c** In vitro development of ookinetes using *P. berghei*-infected blood of control or rPb51-immunized mice. Infected blood on day 3 post *P. berghei* infection collected from these two groups of mice were incubated with culture medium (1:10) and parasite stages were counted at 24 h of incubation. **d** Direct mosquito feeding assay on control and rPb51-immunized mice. For **a**–**d**, means were representative of three separate experiments. Error bars indicate mean ± SD. **Indicate significant difference compared with the control sera (*P* < 0.01)

With Pb51 expression in sexual-stage parasites, the potential TB effect of the anti-rPb51 antibodies using both in vitro and in vivo assays were further investigated. In vitro incubation of the anti-rPb51 antisera with *P. berghei* infected blood at dilutions of 1:5, 1:10 and 1:50 did not have any noticeable effect on the exflagellation of male gametocytes as compared to the control sera (Fig. 5a). However, in vitro culture of ookinetes with the anti-rPb51 antisera, at all dilutions tested, significantly reduced the ookinete numbers by 49.3, 48.7, and 31.0%, respectively, as compared to the control sera (Fig. 5b, *P* < 0.01, Student *t* test). To observe which steps of the ookinete development were obstructed by the anti-rPb51 sera, infected blood was collected from the rPb51-immunized group or the control group on day 3 after infection, when gametocytaemia between the two groups was not statistically different, and was mixed with culture medium at 1:10. At 24 h of the in vitro culture, most parasites (75.4%) in the control group progressed to the mature ookinete stages, whereas 45.4 and 37.4% of the parasites in the medium containing antisera from rPb51-immunized mice were at the retort and ookinete stages, respectively, leading to a 38% reduction of mature ookinetes (Fig. 5c, *P* < 0.01, Student *t* test).

To further examine the TB effect of anti-rPb51 antibodies in vivo, immunized mice were used in DFA. Compared to the control group (immunization with the control protein), mosquitoes fed on rPb51-immunized mice showed a significant reduction in both infection prevalence and oocyst intensity (Table 1 and Fig. 5d). Whereas the average infection prevalence in mosquitoes fed on the control mice was 96%, it was reduced to 74.7% in mosquitoes fed on rPb51-immunized mice (Table 1, *P* < 0.001, Fisher’s exact test). Further, mosquitoes fed on control mice displayed a mean oocyst intensity of 86.7/midgut, whereas it was reduced to 31.9/midgut in mosquitoes fed on the rPb51-immunized mice (Table 1, Fig. 5d, *P* < 0.001, Mann–Whitney *U* test).

**Table 1:** Transmission-blocking effects of mouse antiserum produced by rPb51 immunization

	Control mice			rPb51 immunized mice		
	Con-M1	Con-M2	Con-M3	rPb5-M1	rPb5-M2	rPb5-M3
Mosquitoes infected/dissected	47/50	49/50	48/50	38/50	39/50	35/50
Prevalence of infection (%)^a^	94	98	96	76	78	70
Mean prevalence (%)	96			74.7		
Reduction in prevalence (%)^b^						21.3*
Oocyst intensity^c^	87.2	90.1	82.9	33.8	29.9	32.1
SEM^d^	6.7	6.7	6.8	4.5	3.6	4.4
Mean oocyst intensity			86.7			31.9*
Reduction in oocyst intensity (%)^e^						54.8

* *P* < 0.001 for comparisons between the experimental group and the control group

^a^The prevalence of infection was calculated by the number of mosquitoes with oocysts/total mosquitoes dissected in each group × 100%

^b^The percent reduction of prevalence was calculated as % mean prevalence_control_ − % mean prevalence _rPb51_

^c^Mean number of oocysts per mosquito midgut

^d^Standard error of the mean

^e^The percent reduction in oocyst intensity was calculated as (mean oocyst intensity_control_ − mean oocyst intensity_rPb51_)/mean oocyst intensity_control_ × 100%

## Discussion

The development of an effective malaria vaccine is important for the control and eventual elimination of malaria in endemic areas [32]. The publication of the genomes, transcriptomes and proteomes of a number of malaria parasite species [33–35] has enabled in silico identification of potential malaria vaccine candidates [36]. In this study, a conserved *Plasmodium* membrane protein Pb51 that is expressed in both asexual blood stages and sexual stages was identified. The partial domain of Pb51 protein was expressed and raised polyclonal antisera in mice, which were found not only to provide protection against *P. berghei* blood stages but also to possess effective TB activity.

Development of an effective malaria vaccine depends on better understanding of the parasite biology and the host immune responses [37]. Malaria parasites have a complex life cycle and sub-unit vaccines containing multiple components and targeting multiple stages have been pursued. One strategy to achieve this goal is to express fusion proteins of different antigens. For example, a fragment of the asexual blood-stage antigen glutamate-rich protein (GLURP) fused with a functional fragment of the sexual stage antigen Pfs48/45 (10 C) can induce antibodies showing both asexual growth inhibition and TB activities against *P. falciparum* [21]. Additional combinations have also been tried including sporozoite, asexual blood stage and sexual stage antigens (PfTRAP, PfCelTos, PfCSP, PfMSP1-19, PfMSP4, PfMSP8, PfMSP8, PfMSP3, Pfs230, and Pfs25); some of these combinations showed great promise [38]. However, multiple unrelated component domains in one recombinant protein are not always compatible with each other, which may lead to misfolding and aggregation of the protein that impair their biological activity [39]. In addition, the selection of suitable linkers between the domains is another difficult factor [40]. The protein expression profile of P51 in both asexual blood stages and sexual stages naturally circumvents this problem. Though P51 contains an OST3_OST6-like domain that suggests localization in the endoplasmic reticulum, the inclusion of a signal peptide and PEXEL motif suggests that this protein is likely exported to the RBC and potentially its membrane. Here the evidence show that Pb51 is indeed expressed in multiple stages and localized primarily on the outer membranes of iRBCs except for the ring stage. Antibodies induced by the rPb51 not only inhibited the parasite proliferation during the asexual erythrocytic cycle, but also inhibited the formation of ookinetes and subsequent transmission to the mosquitoes. Since the protection activities observed in this study were the results of immunization of mice with a Pb51 fragment, which contains only limited epitopes, future studies using full-length Pb51 may provide better protective activity. Exploration of eukaryotic protein expression systems may offer further improvement of antigenicity of the recombinant protein. Furthermore, because Freund’s adjuvants are unsuitable for human use, future investigations of the P51 vaccine potential in human malaria parasites using an adjuvant suitable for clinical development (e.g., Montanide ISA-51 or Alhydrogel) are warranted.

## Conclusions

This study identified a conserved *Plasmodium* protein P51, which was expressed in all asexual erythrocytic stages (rings through schizonts) and in sexual stages (gametocytes, zygotes, retorts, ookinetes and sporozoites) of *P. berghei.* The rPb51 possesses excellent immunogenicity and antibodies against this protein inhibited both asexual proliferation in RBCs as well as transmission of the parasites to the mosquitoes. Altogether, these data support further assessment of P51 as a potential candidate for malaria vaccine development.

## Additional files

**Additional file 1: Figure S1.** Predicted B cell epitopes of the Pb51 protein.

**Additional file 2: Figure S2.** Indirect immunofluorescence assay of Pb51. Different development stages of time points of *P. berghei* were used including schizonts, gametocytes, zygotes, retorts, ookinetes and sporozoites (FITC-green). Cells were proceeded directly for antibody binding. Except for the ring stage where fluorescence was restricted to the parasite inside the iRBC, IFA without membrane permeabilization showed the similar fluorescence patterns as those with membrane permeabilization by treatment with Triton X-100 (Fig. 3b). Nuclei were stained with DAPI (blue). BF: bright field. The scale bar indicates 5 µm.

**Additional file 3: Figure S3.** Effects of passive received anti-rPb51 sera on asexual proliferation, gametocytogenesis and host survival. **a** The group that passively received sera from control group exhibited 1.52-fold higher parasitaemia than the rPb51-immunized group on day 9 post-infection. **b** Gametocytaemia in mice treated with PBS or passively received control sera or anti-rPb51 sera. **c** The group passively received anti-rPb51 sera survived for 4 days longer than the control group, and 5 days longer than the group passively received control sera injected with PBS. For a-c, the data represent results from three separate experiments. Error bars indicate mean ± SD. *P< 0.05, **P< 0.01 (Student’s *t* test).

## Abbreviations

TBV: transmission-blocking vaccine
IFA: indirect immunofluorescence assay
ACT: artemisinin-based combination therapy
CSP: circumsporozoite protein
AMA-1: apical membrane antigen 1
MSP1–3: merozoite surface proteins 1–3
GLURP: glutamate-rich protein
OST complex: oligosaccharyl transferase complex
CFA: complete freund adjuvant
iRBCs: infected red blood cells
aa: amino acid
ELISA: enzyme-linked immunosorbent assay
PBS: phosphate-buffered saline
PBS-T: 0.05% Tween 20 in phosphate-buffered saline
TBS-T: 0.1% Tween 20 in Tris-buffered saline
ip: intraperitoneally
RBCs: red blood cells
