# Supplementary material for: Characterization of Pb51 in Plasmodium berghei as a malaria vaccine candidate targeting both asexual erythrocytic proliferation and transmission
Source: Malar J. 2017 Nov 13;16:458. doi: 10.1186/s12936-017-2107-2 (PMC5683326; doi:10.1186/s12936-017-2107-2)
Supplement: Supplementary file 1 — Additional file 1: Figure S1. Predicted B cell epitopes of the Pb51 protein. [file 12936_2017_2107_MOESM1_ESM.docx]

**Fig. S1** Predicted B cell epitopes of the Pb51 protein (http://imed.med.ucm.es/Tools/antigenic.pl).

**Antibody Epitope Prediction**


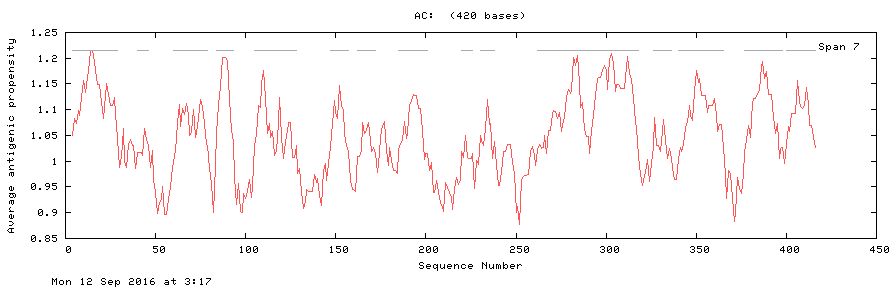


| **Antigenic determinants prediction** |
| --- |
| \| **N** \| **Start** \| **Sequence** \| **End** \| \| --- \| --- \| --- \| --- \| \| 1 \| 4 \| TYSKLYFFFLYFVIVFFKSVTYCLND \| 29 \| \| 2 \| 40 \| INKKIKL \| 46 \| \| 3 \| 60 \| EISFHYIHELSLSDYIDYVL \| 79 \| \| 4 \| 84 \| EYDCVLFLID \| 93 \| \| 5 \| 105 \| RKTYVLLELFNTVAKKIILENIFL \| 128 \| \| 6 \| 147 \| LLKPIFFFYVN \| 157 \| \| 7 \| 162 \| NLNPLKHIHMI \| 172 \| \| 8 \| 185 \| TLYNHKYYSLIYSIYML \| 201 \| \| 9 \| 220 \| IQIELNI \| 226 \| \| 10 \| 230 \| TNYFLKFIY \| 238 \| \| 11 \| 262 \| IFLNIKMYLSFIIGIIFMLLYLFILIVNKYNIIIFICSYILYFICLSGLFHCLIYQS \| 318 \| \| 12 \| 326 \| TLDSILNTYIY \| 336 \| \| 13 \| 340 \| NSQYIYEGLFVSFLIFIISFSLFILN \| 365 \| \| 14 \| 377 \| LNFFFFFFLIFIICISLNIIHK \| 398 \| \| 15 \| 400 \| NTYKVYFSTYVFFPPIK \| 416 \| |
