# Supplementary figures and images for: Characterization of Pb51 in Plasmodium berghei as a malaria vaccine candidate targeting both asexual erythrocytic proliferation and transmission
Source: Malar J. 2017 Nov 13;16:458. doi: 10.1186/s12936-017-2107-2 (PMC5683326; doi:10.1186/s12936-017-2107-2)

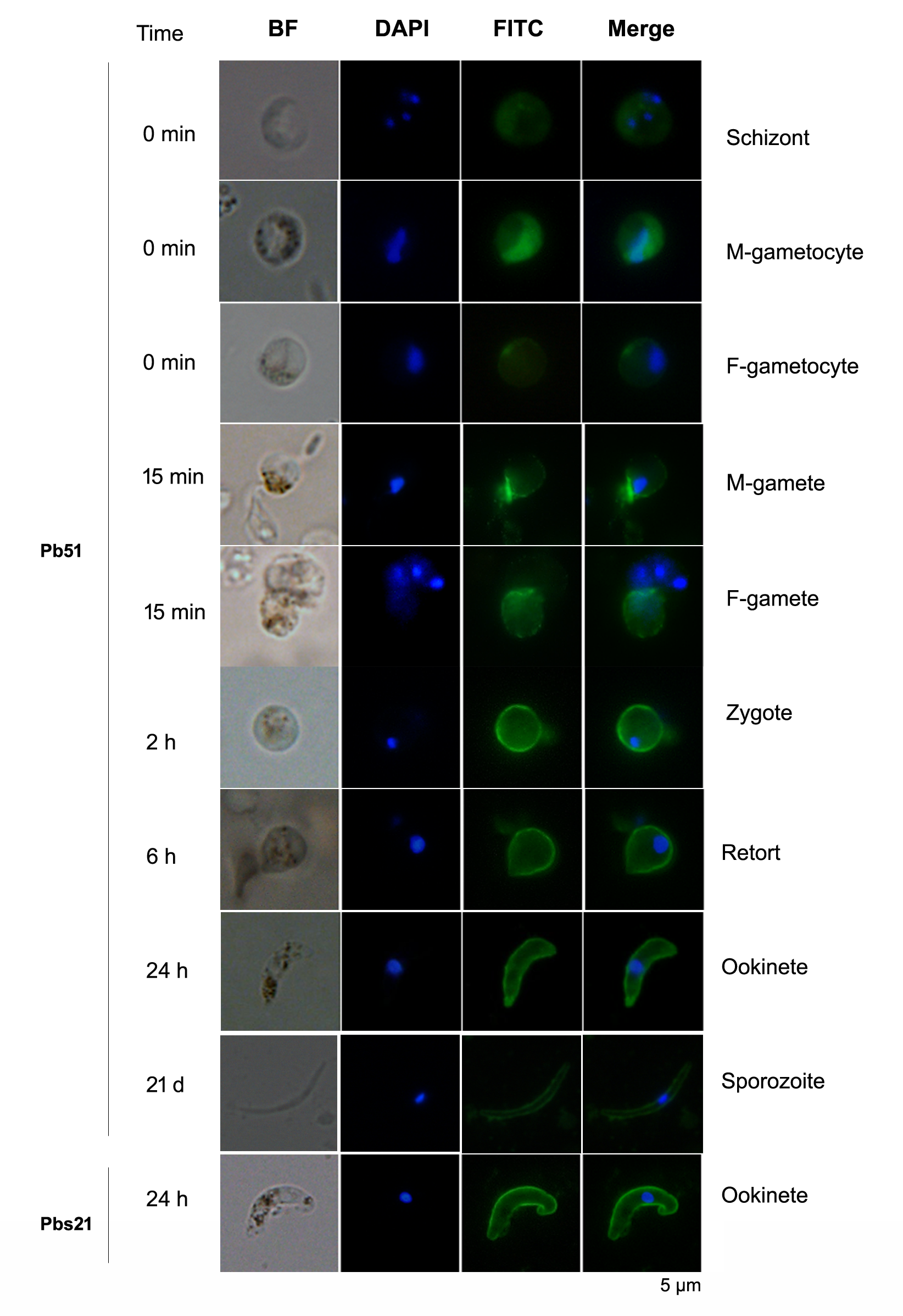

Supplement: Supplementary file 2 — Additional file 2: Figure S2. Indirect immunofluorescence assay of Pb51. Different development stages of time points of P. berghei were used including schizonts, gametocytes, zygotes, retorts, ookinetes and sporozoites (FITC-green). Cells were proceeded directly for antibody binding. Except for the ring stage where fluorescence was restricted to the parasite inside the iRBC, IFA without membrane permeabilization showed the similar fluorescence patterns as those with membrane permeabilization by treatment with Triton X-100 (Fig. 3b). Nuclei were stained with DAPI (blue). BF: bright field. The scale bar indicates 5 µm. [file 12936_2017_2107_MOESM2_ESM.tif]

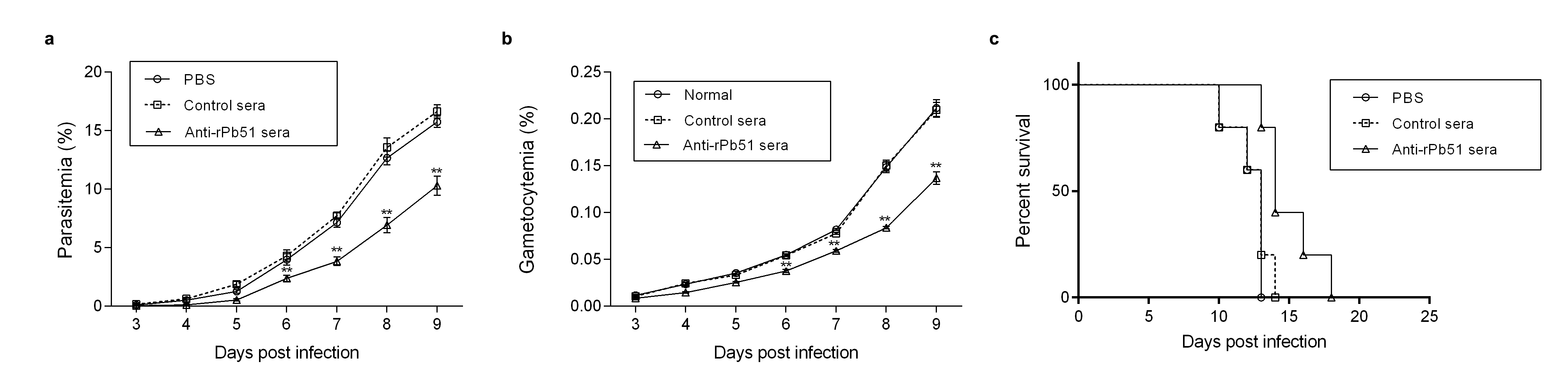

Supplement: Supplementary file 3 — Additional file 3: Figure S3. Effects of passive received anti-rPb51 sera on asexual proliferation, gametocytogenesis and host survival. a The group that passively received sera from control group exhibited 1.52-fold higher parasitaemia than the rPb51-immunized group on day 9 post-infection. b Gametocytaemia in mice treated with PBS or passively received control sera or anti-rPb51 sera. c The group passively received anti-rPb51 sera survived for 4 days longer than the control group, and 5 days longer than the group passively received control sera injected with PBS. For a-c, the data represent results from three separate experiments. Error bars indicate mean ± SD. *P< 0.05, **P< 0.01 (Student’s t test). [file 12936_2017_2107_MOESM3_ESM.tif]
